# Liver X receptor agonist T0901317 alleviates sepsis-induced acute lung injury by enhancing macrophage autophagy

**DOI:** 10.3389/fphar.2025.1552034

**Published:** 2025-09-09

**Authors:** Ben Wang, Ran Wang, Xueling Wu, Yu Zhong, Chaowang Huang, Zhi Xu, Liang Guo

**Affiliations:** ^1^ Department of Obstetrics and Gynecology, Daping Hospital, Army Medical University (Third Military Medical University), Chongqing, China; ^2^ Department of Respiratory and Critical Care Medicine, Second Affiliated Hospital of Third Military Medical University (Army Medical University), Chongqing, China; ^3^ Department of Respiratory Medicine, Renji Hospital, Shanghai Jiaotong University School of Medicine, Shanghai, China; ^4^ Department of Nephrology, Southwest Hospital Jiangbei Area (The 958th Hospital of the Chinese People’s Liberation Army), Chongqing, China; ^5^ Department of Geriatrics and Special Services Medicine, Xinqiao Hospital, The Second Affiliated Hospital, Army Medical University, Chongqing, China

**Keywords:** liver X receptor alpha, liver X receptor agonist T0901317, acute lung injury, autophagy, S100A8

## Abstract

**Background:**

This study was aimed at identifying the effects of liver X receptor alpha (LXRα) on sepsis-induced acute lung injury (ALI) and clarifying its novel regulatory mechanisms using bioinformatics and experimental methods.

**Methods:**

Bioinformatics analysis of the differentially expressed genes and functional annotations were performed. Lipopolysaccharide (LPS) was administered intraperitoneally for sepsis-induced ALI in a mouse model; then, the LXR agonist T0901317 (T0) was administered to the mice along with RAW264.7 macrophages for LXRα activation. We then performed hematoxylin and eosin staining, estimated the total protein in the bronchoalveolar lavage fluid, and detected the expressions of TNFα and IL6 by reverse transcription polymerase chain reaction to evaluate the inflammatory injury in the lung tissues. Autophagy was detected via immunohistochemistry, transmission electron microscopy, and Western blotting. RNA sequencing was then used to analyze the autophagy-related genes regulated by LXRα, and the cells were transfected with S100A8-siRNA to determine whether LXRα regulated inflammatory damage by regulating the autophagy-related gene S100A8. The clinical correlation between LXRα and S100A8 was determined through analysis of human transcriptome data.

**Results:**

The bioinformatics analyses revealed that LXRα (NR1H3) was downregulated in sepsis-induced ALI models and that LXRα might regulate autophagy. The animal- and cell-based experiments further verified these findings. The LXR agonist T0 was found to alleviate lung damage and reduce the expressions of inflammatory factors in the lung tissues and cells. After inhibiting autophagy with 3-methyladenine, the protective effects of T0 on inflammatory damage were shown to be inhibited. Subsequently, RNA sequencing of the macrophages was performed, and four genes (*ABCG1*, *FASN*, *S100A8*, and *SNORD118*) were obtained by intersection of the upregulated and downregulated differential genes with the autophagy gene set. However, among these genes, only *S100A8* that was increased in ALI and decreased markedly after T0 treatment exhibited a negative correlation with T0. Following *S100A8* knockdown in the macrophages with S100A8-siRNA, the IL-6 expression was noted to decrease in cells treated with T0+LPS+S100A8-siRNA than those treated with LPS+T0. Analysis of the human transcriptome data revealed a significant negative correlation between LXRα and S100A8 (R = −0.98, *p* < 0.001).

**Conclusion:**

The findings of this study suggest that T0 attenuates sepsis-induced pulmonary injury by promoting macrophage autophagy via suppression of S100A8 expression.

## Introduction

Acute lung damage is a result of various internal and external influences on the lungs, such as infection, sepsis, or trauma, and usually occurs owing to an overall inflammatory reaction (Li et al., 2021; Chen et al., 2021; Wheeler and Bernard, 2007). The presence of such damage often leads to severe acute respiratory distress syndrome (ARDS), which is characterized by sudden onset, rapid deterioration, and poor outcome. These factors significantly contribute to the high fatality rate of ARDS, which ranges between 30% and 40% (Meyer et al., 2021). Despite efforts to treat any underlying diseases using mechanical ventilation along with vasodilators, surfactants, antioxidants, glucocorticoids, and anti-inflammatory medications, a comprehensive understanding of the intricate nature of acute lung injury (ALI) as well as a definite cure are lacking (Su et al., 2021; Yamashita et al., 2012). Importantly, we must improve our understanding of the drivers of ALI while identifying the possible pathological influences (He et al., 2021). Therefore, bioinformatics analysis is an invaluable tool for exploring the fundamental mechanisms underlying sepsis-induced ALI while pinpointing new targets for prospective clinical use.

Pulmonary lipids maintain the structural integrity and operational efficacy of the lungs (Zhou et al., 2023). Some earlier studies have shown that disorders in lipid metabolism are associated with ALI (Du et al., 2020; Chen et al., 2024a). We employed bioinformatics approaches to explore online datasets and discovered that the lipid biosynthesis process was important for ALI. The liver X receptors (LXRs) are key molecules that regulate lipid metabolism; they are further classified into LXRα and LXRβ subtypes and have been identified in the context of inflammation regulation (Lalloyer et al., 2009; Liu et al., 2012a; Joseph et al., 2003). The expression of LXRα was reduced in individuals with sepsis-induced lung injury, as demonstrated by bioinformatics methods using online databases. Hence, we hypothesized that LXRα might regulate ALI.

As an essential mechanism of cell renewal and protection, autophagy is regarded as a crucial factor that regulates inflammation and lipid biosynthetic processes (Matsuzawa-Ishimoto et al., 2018; Zhong et al., 2022). Experiments have demonstrated that autophagy is tightly related to ALI and can serve as an important target for therapeutic interventions in ALI (Vishnupriya et al., 2020; Hu et al., 2014). Additionally, LXRα has been reported to enhance autophagy and safeguard the myocardial cells from inflammatory injuries (Peng et al., 2014). Nevertheless, the precise mechanisms by which LXRα modulates autophagy in the context of ALI remain unclear.

Given the significant roles of alveolar macrophages in ALI (Cheng et al., 2021; Kumar, 2020) as well as the discovery that LXRα was highly expressed in macrophages and was closely associated with autophagy, as demonstrated by bioinformatics methods using online databases, we hypothesize that LXRα may regulate sepsis-induced lung injury through autophagy. Consequently, in our study, we used the liver X receptor antagonist T0901317 (T0) to enhance the expression of LXRα and further verify the functions of LXRα in sepsis-induced lung tissue; further, we identified a novel mechanism by which LXRα regulated macrophage autophagy via the S100A8 signaling pathway, which we hope would serve as a novel target for treating ALI (Supplementary Figure S1).

## Materials and methods

### Data sources and analysis

The gene expression profiles of GSE262393 and GSE269740 were fetched from the Gene Expression Omnibus (GEO) database using the keyword “acute lung injury (ALI).” Here, GSE269740 represents the expression profile of bulk RNA sequencing (RNA-seq) data from the ALI mouse model, while GSE262393 contains single-cell RNA-seq data from the normal lungs of a mouse model. The gene expression profiles were subsequently preprocessed and normalized using R software (version 4.2.1). Additional gene expression profiles (GSE21837 and GSE10474) were retrieved from the GEO database to supplement the clinical correlation analysis of LXRα and S100A8 expressions. Here, GSE21837 represents the expression profiling of bulk RNA-seq data of normal persons, and GSE10474 represents the expression profiling of bulk RNA-seq data of ALI patients.

### Analysis of differentially expressed genes (DEGs) and functional annotations

The R package “limma” (version 3.42.2) was used to analyze the DEGs between the ALI and normal samples (Ritchie et al., 2015). The default thresholds were a log-transformed fold change greater than 1 (in terms of absolute value) along with an adjusted *p*-value of less than 0.05 after Bonferroni’s correction. To conduct the pathway enrichment analyses of the DEGs, we used Metascape with the default parameter settings. The pathway enrichment analyses of the upregulated and downregulated DEGs were conducted independently using Metascape. Each enriched functional term was considered a node, and pairs of nodes were connected when the kappa similarity score was greater than 0.3. Additionally, the “clusterProfiler” R package (version 4.8.3) was employed for the gene set enrichment analysis to identify specific enriched biological processes and/or signaling pathways.

### Identification of core autophagy- and lipid-associated genes via bioinformatics analyses

The autophagy-related expression genes were explored and selected from GeneCards, which is a searchable and integrative database. The autophagy-associated genes were first extracted from GeneCards, and genes with relevance scores ≥1 were selected. The lipid-associated genes were then extracted from GeneCards, and genes with relevance scores ≥5 were selected. These results were intersected with those of the DEGs, and the core autophagy-associated genes were then analyzed through experimental verification.

### Dimensionality reduction, clustering, and annotation of the scRNA-seq data

The matrix of identified data was transformed into a Seurat object using the Seurat package (version 4.2.0), and quality filtering was performed based on specific sequencing characteristics. The general approach used here was the same as that described previously (Chen et al., 2022).

### RNA-seq process

Once the samples were collected and processed, the mRNA was isolated and concentrated from the total RNA by exploiting the distinct structural features of mRNAs or by employing specialized mRNA enrichment kits. The mRNA was subsequently fragmented to enable reverse transcription and sequencing. The resulting libraries were then sequenced using high-throughput sequencing instruments with the DNBSEQ-T7 sequencing platform. The sequencing strategy was selected according to the specific demands for sequencing read lengths and data volumes.

### Construction of animal models and experimental protocols

Eight-week-old male C57BL/6 mice were provided by Xinqiao Hospital laboratory center at the Army Medical University, whose ethics committee also approved this study (no. AMUWEC20235039). To determine the effects of T0 on ALI *in vivo*, the mice were randomly divided into four groups as dimethylsulfoxide (DMSO)-treated mice (control), lipopolysaccharide (LPS)-treated mice, T0-treated mice, and T0+LPS-treated mice (n = 3). T0 was administered to the mice in accordance with methods established in literature previously (Peng et al., 2014). Briefly, from days 1 to 5, the LPS+T0- and T0-treated mice were intraperitoneally administered T0 dissolved in DMSO at the dose of 50 mg/kg (Cayman Chemical Company, Ann Arbor, MI, United States). Moreover, the LPS- and DMSO-treated mice were intraperitoneally administered DMSO in a volume equivalent to that of T0. On day 6, LPS (15 mg/kg) (*Escherichia coli* O55:B5, Sigma, St. Louis, MO, United States) was administered to the LPS- and T0+LPS-treated mice, while the DMSO- and T0-treated mice were injected with saline. Six hours after the LPS and saline treatments, samples were collected from the mice, and the left lung of each mouse was used for hematoxylin and eosin (H&E) staining. Then, the right lung from each mouse was used for Western blotting and reverse transcription polymerase chain reaction (RT-PCR).

### Cell culture, drug treatment, and small interfering RNA (siRNA) transfection

The cell samples were separated into four groups to explore the effects of T0 on lung injury, namely DMSO-treated (control), LPS-treated, T0-treated, and T0+LPS-treated groups. The RAW264.7 cells were treated after they reached 70% confluence. Next, the cells were transfected with 600 nM of S100A8-siRNA (General Biosystems, Hefei, China) and control-siRNA using an advanced DNA and RNA transfection reagent (Genlantis, San Diego, CA, United States). After 6 h, the cells were co-incubated with 0.01 mM of T0 and/or 3 mM of 3-methyladenine (3-MA) for 12 h. Lastly, the cells were co-incubated with LPS (10 μg/mL) and harvested after 24 h.

### RNA isolation and quantitative real-time polymerase chain reaction (qRT-PCR)

RNAs were collected from the lung tissues and RAW264.7 cells for qRT-PCR. The reaction conditions were as follows: 95 °C for 30 s, followed by 40 cycles at 95 °C for 5 s, and 60 °C for 34 s. GAPDH was used as the internal reference, and data analysis was performed using the comparative double-delta Ct method with the mRNA levels of interest normalized to the GAPDH levels. The primers used were as follows: TNFα: forward: 5′-TCT​CTT​CAA​GGG​ACA​AGG​CT-3′, reverse: 5′-GGC​AGA​GAG​GAG​GTT​GAC​TT-3’; S100A8: forward: 5′-AAA​TCA​CCA​TGC​CCT​CTA​CAA​G-3′, reverse: 5’-CCC​ACT​TTT​ATC​ACC​ATC​GCA​A-3’; GAPDH: forward: 5′-AAC​TTT​GGC​ATT​GTG​GAA​GG-3′, reverse: 5′-ACA​CAT​TGG​GGG​TAG​GAA​CA-3’; IL6: forward: 5′-CTC​TGG​GAA​ATC​GTG​GAA​AT-3′, reverse: 5′-CCA​GTT​TGG​TAG​CAT​CCA​TC-3’.

### Western blotting

The cell proteins were collected using ice-cold RIPA lysis buffer and then used to detect the expressions of LXRα (1:1,000, Abcam, Cambridge, United Kingdom), NF-kB p65 (1:500, Beijing Golden Bridge Biotech), and LC3B (1:1,000, Cell Signaling Technology, Danvers, MA, United States). Here, electrophoresis, membrane transfer, and blocking were performed by the usual procedures. The primary antibodies were incubated for 24 h at 4 °C, and the homologous secondary antibodies were incubated for 2 h at room temperature. The results were then detected by the enhanced chemiluminescence (ECL) method.

### Histological evaluation and immunohistochemistry (IHC)

The lung tissue slides were placed in 4% paraformaldehyde diluted in phosphate-buffered saline (PBS), embedded in paraffin, dewaxed, and further dehydrated. The dehydrated tissue slides were then stained with H&E, and the lung histology was evaluated according to the “Lung Injury Scoring System” recommended by the 2010 American Thoracic Society Symposium Report (Matute-Bello et al., 2011). The specific scoring criteria were as follows. A: Quantities of neutrophils within alveoli in each high-power field of view: 0 = 0 points, 1–5 = 1 point, and >5 = 2 points. B: Quantities of neutrophils in the interstitial space in every high-power field of view: 0 = 0 points, 1–5 = 1point, and >5 = 2 points. C: Development of pulmonary hyaline membranes in each high-power field of view: none = 0 points, 1 = 1 point, and >1 = 2 points. D: Filling of alveolar protein debris: none = 0 points, 1 = 1 point, and >1 = 2 points. E: Augmentation of alveolar space: <2× normal thickness = 0 points, 2–4× normal thickness = 1 point, and >4× normal thickness = 2 points. An experienced pathologist selected 10 high-power fields of view for lung injury pathological scoring in a double-blind manner. The lung injury score was then calculated as {[(20 × A) + (14 × B) + (7 × C) + (7 × D) + (2 × E)]/(number of fields of view)} × 100. To verify whether the autophagy marker LC3B was expressed in the lung tissues, the samples were incubated with primary antibodies targeting LC3B (1:100, Cell Signaling Technology, Danvers, MA, United States) overnight at 4 °C. The next day, the slides were incubated with anti-rabbit secondary antibody for 2 h at room temperature, followed by three washes with PBS. Lastly, the slides were stained with DAB.

### Total protein in the bronchoalveolar lavage fluid (BALF)

BALF samples were collected as described previously (Chen et al., 2011). The freshly obtained cells were resuspended in PBS containing 1% bovine serum albumin (BSA). The suspension was then centrifuged at 2,000 rpm for 10 min, and the total protein was measured using the BCA assay kit.

### Transmission electron microscopy (TEM)

The cells were fixed using 2.5% glutaraldehyde, followed by post-fixation with 2% osmium tetroxide. The RAW264.7 cells were embedded in a 1 × 1 × 1 mm block and cut into slices of thickness 80–100 nm for TEM observations.

### Statistical methods

All statistical analyses were performed using R software (version 4.2.1). The quantitative data are presented in the form of box plots, in which the boxes signify the interquartile ranges, whiskers extend to ±1.5 interquartile ranges, dots denote the outliers, and bold lines represent the median values. The statistical significance was ascertained by the hypergeometric test; *p*-values and adjusted *p*-values lower than 0.05 were regarded as statistically significant. The data were statistically analyzed using SPSS software (version 22.0) and presented as mean ± standard error of the mean (SEM) values. ANOVA or Student’s t-test was used to evaluate differences between groups, and the statistical significance was considered at *p* < 0.05.

## Results

### Differential expression analysis and clustering reveal distinct expression patterns in ALI samples

**FIGURE 1:**
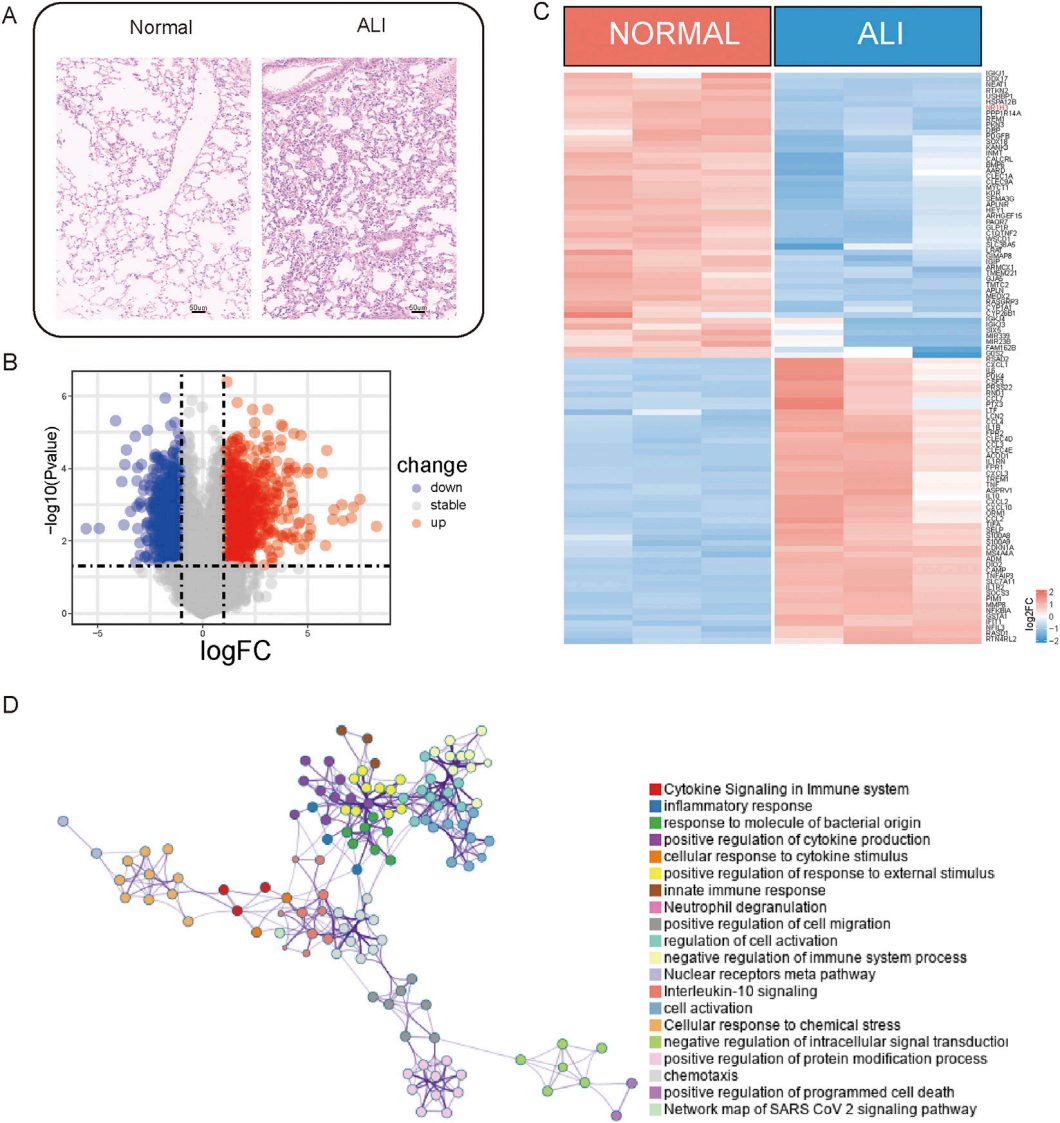
Upregulated genes associated with inflammation. **(A)** Lung tissues from acute lung injury (ALI) mice and normal controls were subjected to immunohistochemistry (IHC) staining. **(B)** Volcano plot illustrating the differentially expressed genes (DEGs) between the ALI and normal samples. **(C)** Heatmap displaying the expression levels of the DEGs between ALI and normal samples. **(D)** Network of enriched terms across the upregulated DEGs between ALI and normal samples.

A differential expression analysis was performed on three normal and three ALI samples from the GSE269740 dataset using the R package *limma* (version 3.58.1) to analyze the DEGs between these samples (Figure 1). The analysis revealed a total of 1,972 DEGs, with 941 upregulated and 1,031 downregulated genes (*p* < 0.05, |log2 FC| > 1; Figure 1B). The heatmap in the figure illustrates the expression levels of the top-50 DEGs that show the distinct expression patterns between the two groups (Figure 1C). All statistically enriched terms of the upregulated DEGs were subsequently identified using Metascape based on the default choices under express analysis. The pathways enriched for inflammation and immunity, such as cytokine signaling in the immune system, inflammation, cytokine production regulation, and neuron projection development, were identified (Figure 1D).

**FIGURE 2:**
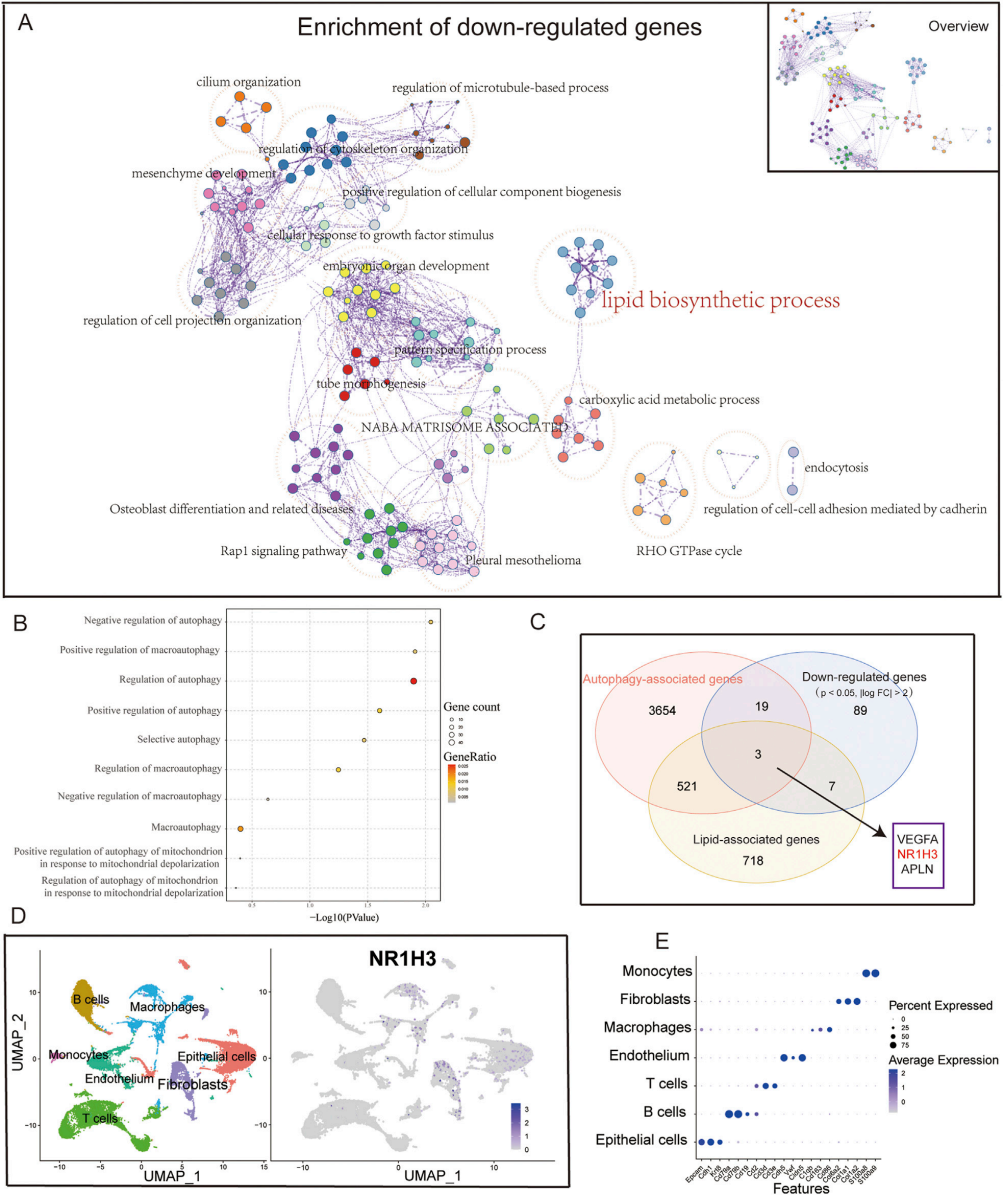
Downregulated genes associated with autophagy. **(A)** Overview of the network of enriched terms across downregulated DEGs between ALI and normal samples. The network was obtained via functional enrichment analysis using Metascape, where each node represents a particular gene ontology (GO) term and similar terms are clustered and merged for annotation. The connections between the nodes reflect protein‒protein interactions. **(B)** Dot plot showing the autophagy-associated pathways in the GO enrichment analysis of DEGs. **(C)** Venn diagram showing the close relationship between downregulated genes and autophagy. **(D)** UMAP plot of the cells colored by cell type and **(E)** feature plot of *NR1H3* expressions in three samples of normal lung tissue.

A comprehensive enrichment network was generated to represent the enriched functional terms for the downregulated DEGs (Figure 2A), which revealed associations with lipid biosynthetic and carboxylic acid metabolic processes. Upon intersecting the autophagy-related gene set with the downregulated genes (*p* < 0.05, |log2 FC| > 1), the autophagy-related genes were noted to account for a significant portion of the downregulated genes (approximately 41.9%). Finally, the selected autophagy-related gene ontology (GO) pathways involving both upregulated and downregulated genes were combined for further analyses (Figure 2B). Given that the downregulated genes are related to lipid metabolism and autophagy, we considered the intersection of the downregulated DEGs (*p* < 0.05, |log2 FC| > 1), autophagy gene set, and lipid metabolism gene set to obtain three important genes (*VEGFA*, *NR1H3*, and *APLN*) (Figure 2C). By combining our previous research findings and referring to literature (Hernández-Hernández et al., 2024), we hypothesized that LXRα (NR1H3) may play a relatively important role in ALI. Here, LXRα is noted as a gene with dual functions in lipid regulation and autophagy. Very few studies have investigated the effects of this gene on lung-injury-related autophagy. Our aim was to investigate whether LXRα could affect ALI by modulating autophagy. Single-cell sequencing data from GSE262393 revealed that *NR1H3* was predominantly expressed in macrophages, epithelial cells, and fibroblasts (Figures 2D,E). These findings prompt us to hypothesize that LXRα may regulate sepsis-induced lung injury through its effects on macrophage autophagy.

### Verification of LXRα expressions in animals and cells

**FIGURE 3:**
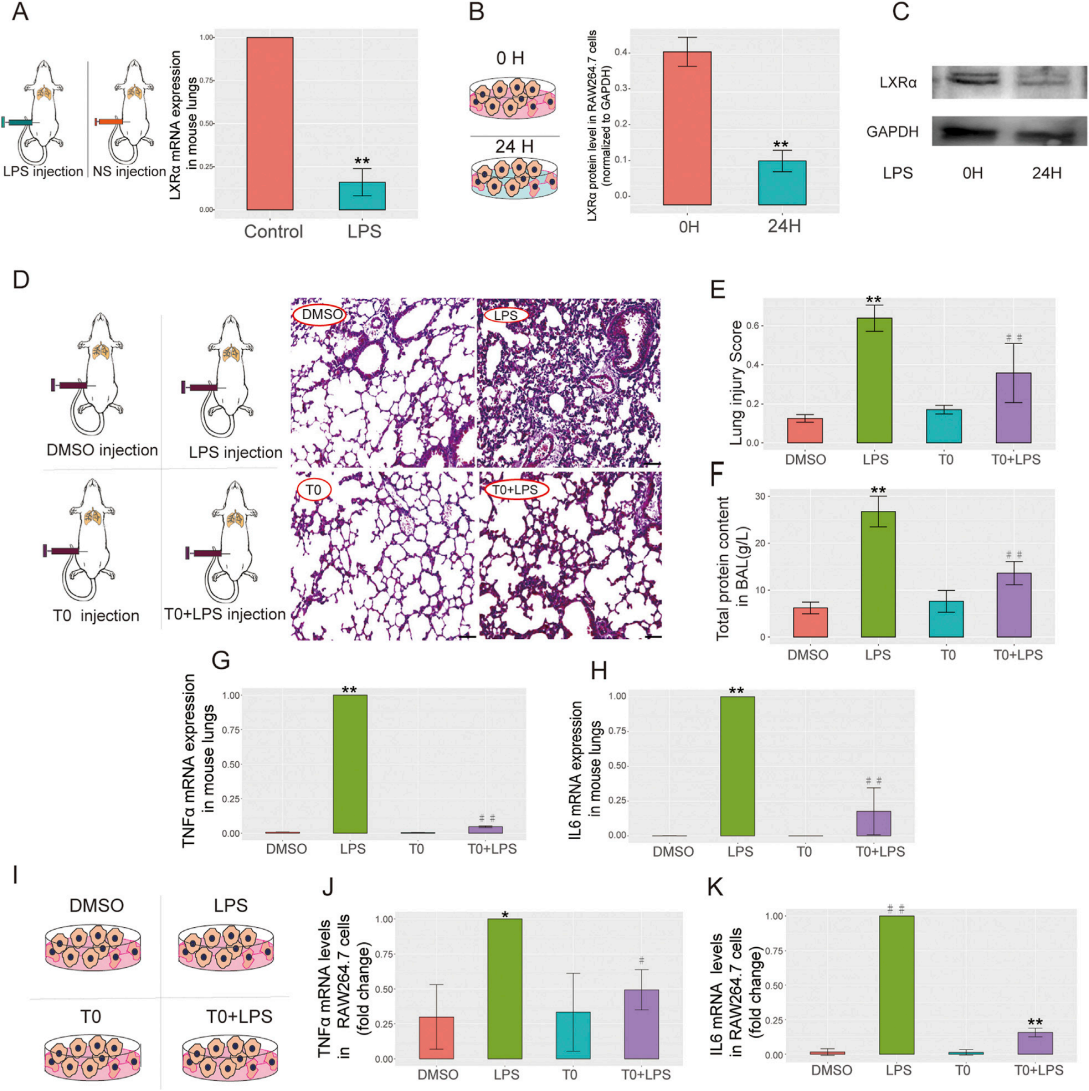
LXRα expressions in a mouse model of ALI, RAW264.7 cells, and T0-treated ALI. **(A)** Lipopolysaccharide (LPS; 15 mg/kg) was administered to the mice for 6 h and quantitative real-time polymerase chain reaction (qRT-PCR) was performed to measure the LXRα levels in the lung tissues (**p < 0.01). **(B,C)** LPS (10 µg/mL) was administered to RAW264.7 cells for 24 h, and the LXRα protein levels were examined by Western blotting (**p < 0.01). The results are presented as the mean ± standard error of the mean (SEM) values (n = 3). **(D–H)** LPS (15 mg/kg) was administered to the mice on day 6 for 6 h, followed by T0 (50 mg/kg) from days 1 to 5. **(D)** H&E staining was performed to detect pathological changes in the lung tissues. Scale bar = 100 μm. **(E)** Lung injury score (**p < 0.01, LPS vs. dimethylsulfoxide (DMSO); ^##^ *p* < 0.01, LPS vs. T0+LPS). **(F)** Total protein in BALF (**p < 0.01, LPS vs. DMSO; ^##^ *p* < 0.01, LPS vs. T0+LPS). **(G)** qRT-PCR analysis of the TNFα mRNA levels in mouse lung tissues. **(H)** qRT-PCR analysis of the IL6 mRNA levels in mouse lung tissues (**p < 0.01, LPS vs. DMSO; ^##^ *p* < 0.01, LPS vs. T0+LPS). **(I–K)** LPS (10 µg/mL) was administered to RAW264.7 cells for 24 h, followed by T0 (0.01 mM) for 12 h. **(I)** RAW264.7 cells were divided into four groups, including DMSO, LPS, T0, and T0+LPS. **(J)** qRT-PCR analysis of the mRNA levels of TNFα (*p < 0.05, LPS vs. DMSO; ^#^ *p* < 0.05, LPS vs. T0+LPS) and **(K)** IL6 (^##^ *p* < 0.01, LPS vs. DMSO; **p < 0.01, LPS vs. T0+LPS) in RAW264.7 cells.

The LXRα expression levels were measured in the mice and cell lines, which showed downregulated LXRα expression levels in the LPS-treated group compared with the levels in the control cells (Figures 3A–C). These findings are in agreement with our bioinformatics results, suggesting that LXRα might participate in sepsis-related lung injury.

### Increased LXR expression ameliorates LPS-induced ALI

T0 was administered to the lung injury mouse model to investigate the association between LXR expression and ALI. Then, H&E staining was used to evaluate the degree of lung injury, while the inflammatory status was evaluated by detecting the TNFα and IL6 mRNA expressions. In contrast to the control group, LPS administration effectively triggered acute lung tissue injury, which was evidenced by the lung histology according to the “Lung Injury Scoring System” recommended by the 2010 American Thoracic Society Symposium Report. Nevertheless, pretreatment with T0 substantially decreased the lung injury scores, as proven by mitigated tissue damage along with reduced alveolar hemorrhage, alveolar wall thickening, and inflammatory cell infiltration (Figures 3D,E). Additionally, the total protein in the BALF was detected; as shown in Figure 3F, in contrast to the LPS-treated mice, the total protein in the BALF was markedly reduced in the T0+LPS-treated group.

The TNFα and IL6 mRNA levels were detected in the mouse lung tissues. As depicted in Figures 3G,H, in contrast to the LPS-treated mice, the expressions of the TNFα and IL6 mRNAs were markedly lower in the T0+LPS-treated group, suggesting that LXR protects against sepsis-induced lung injury. Consistent with the *in vivo* studies, similar results were obtained with the macrophage lines. As shown in Figures 3I–K, in contrast to the LPS-treated cells, the TNFα and IL6 mRNA expressions were both markedly reduced in the T0+LPS-treated RAW264.7 cells.

### T0 enhances autophagic activity in RAW264.7 cells challenged with LPS

**FIGURE 4:**
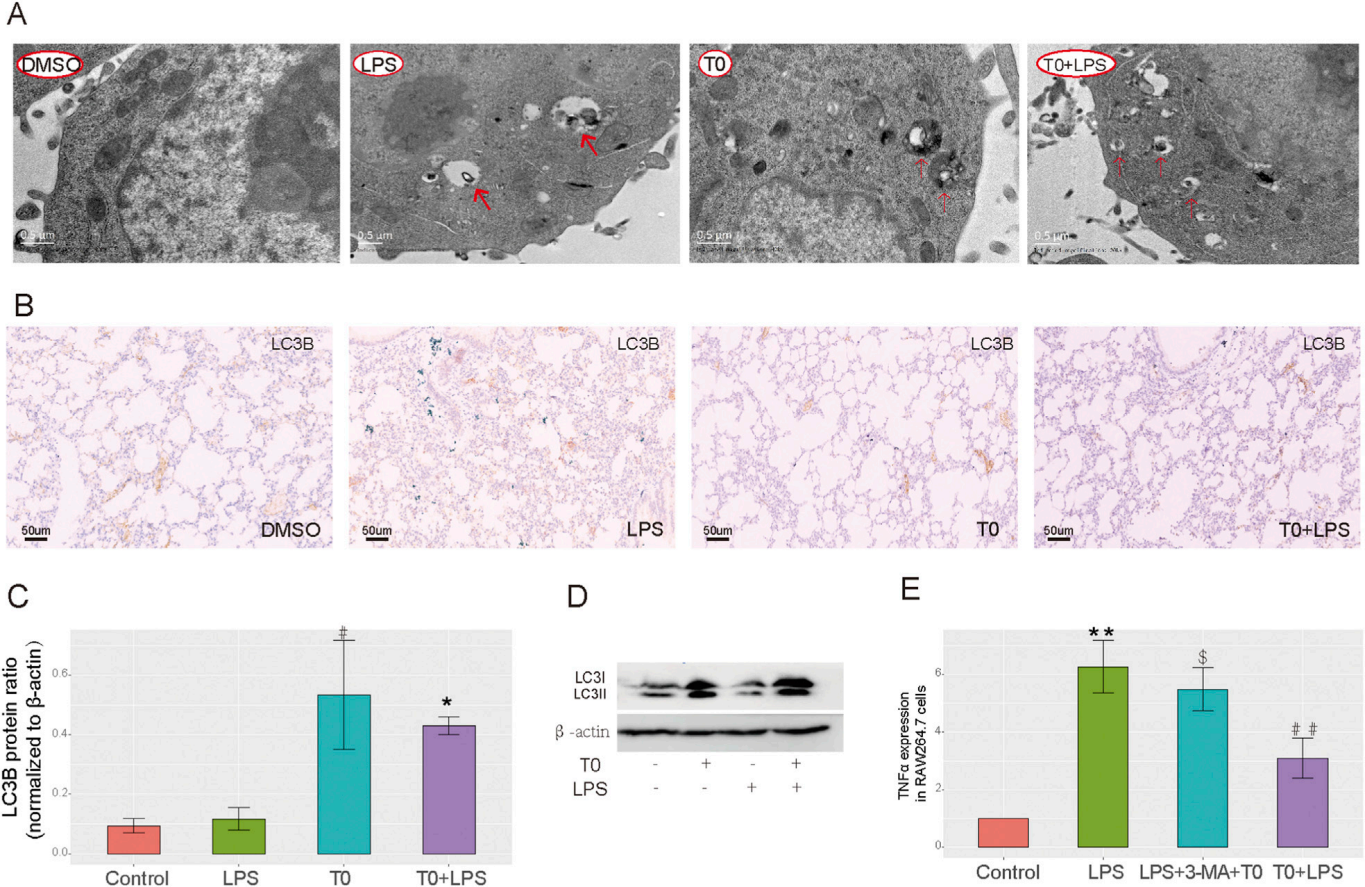
Increased LXR expression improves autophagy. **(A)** Autophagosome levels in the RAW264.7 cells were detected by transmission electron microscopy. **(B)** LC3B expression in lung tissues. Scale bar = 50 μm. **(C)** Quantification of LC3 protein bands (^#^ *p* < 0.05, LPS vs. control; *p < 0.05, LPS vs. T0+LPS). **(D)** LC3B protein levels in RAW264.7 cells using Western blotting. **(E)** qRT-PCR analysis of the TNFα mRNA levels in RAW264.7 cells (**p < 0.01, LPS vs. control; ^##^ *p* < 0.01, LPS vs. T0+LPS; ^$^ *p* < 0.05, T0+3-MA+LPS vs. T0+LPS). The results are presented as mean ± SEM values (n = 3).

TEM was used to detect autophagy in the RAW264.7 cells, which revealed nearly no autophagic vacuoles in the DMSO-treated group, whereas more autophagic vacuoles were observed in both the T0-treated and T0+LPS-treated cells (Figure 4A). The LC3-II/β-actin ratio is widely utilized as an indicator of autophagosome formation (Liu et al., 2012b). As depicted in Figure 4B, the LC3B expressions were higher in the LPS-treated mice than the DMSO-treated group. Moreover, LC3B expressions in the T0+LPS-treated mice were higher than in the LPS-treated mice. As shown in Figures 4C,D, LC3-II expressions were also increased in the T0-treated cells compared to the DMSO-treated group. Moreover, LC3-II expressions were higher in the T0+LPS-treated cells than in the LPS-treated cells. We inhibited autophagy by administering 3-MA and detected the mRNA level of the inflammatory factor TNFα to further clarify whether LXR protects against sepsis-induced ALI by enhancing autophagy. As shown in Figure 4E, the TNFα mRNA expression increased in the T0+LPS+3-MA-treated cells than the T0+LPS-treated group; these results suggest that T0 enhances autophagy in cells challenged with LPS.

### Identification of *S100A8* by RNA-seq of the groups

**FIGURE 5:**
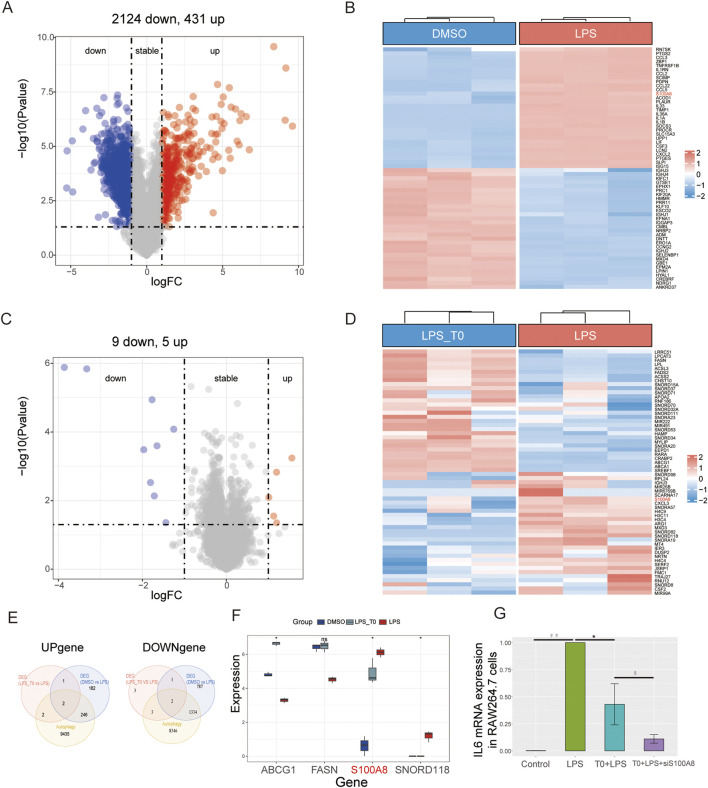
Experimental verification of the key autophagy-related genes. **(A,B)** Volcano plot and heatmap illustrating the DEGs between the DMSO and LPS groups. **(C,D)** Volcano plot and heatmap illustrating the DEGs between the LPS+T0 and LPS groups. **(E)** Venn diagram showing the common genes between autophagy-related genes and DEGs. **(F)** Distribution of the four overlapping genes between the three groups. *p < 0.05; ns, non-significant (*p* > 0.05). **(G)** RAW264.7 cells were transfected with S100A8-siRNA (600 nM) and control-siRNA using an advanced DNA and RNA transfection reagent. After 6 h, the cells were co-incubated with T0 (0.01 mM) for 12 h. Lastly, the cells were co-incubated with LPS (10 μg/mL) and harvested after 24 h. qRT-PCR analysis of the IL6 mRNA levels in the RAW264.7 cells (*p < 0.05, LPS vs. T0+LPS; ^##^ *p* < 0.01, LPS vs. control; ^$^ *p* < 0.05, T0+LPS+S100A8-siRNA vs. T0+LPS). The results are presented as mean ± SEM values (n = 3).

In contrast to the DMSO (control) group, the LPS group showed 431 upregulated and 2,124 downregulated genes (Figures 5A,B). Compared with the LPS+T0-treated cells, the LPS group presented five upregulated and nine downregulated genes (Figures 5C,D). We intersected both the upregulated and downregulated genes with the autophagy-related gene set and acquired four common genes (*S100A8*, *SNORD118*, *ABCG1*, and *FASN*) (Figure 5E). Finally, in the LPS+T0-treated cells, only the *S100A8* gene expression was found to have decreased compared to the LPS group (Figure 5F). A review of relevant literature further revealed that *S100A8* facilitates the progression of lung injury induced by LPS (Fan et al., 2024; Yu et al., 2023). Hence, we postulated that T0 might increase macrophage autophagy to safeguard against lung injury by suppressing *S100A8*.

The S100A8-siRNA was transfected into RAW264.7 cells to interfere with *S100A8* expression and to validate the connection between LXRα and *S100A8* in ALI. The transfection efficiency was then confirmed (Supplementary Figure S2). As shown in Figure 5G, the qRT-PCR analysis revealed that IL6 expression levels in the LPS-treated cells were elevated compared to those in the control group. Nevertheless, the IL6 levels in the T0+LPS-treated cells were higher than those in the T0+S100A8-siRNA+LPS cells. Collectively, we demonstrated that activated T0 could partly increase autophagy by suppressing *S100A8*.

### Bioinformatics analysis reveals clinical correlation between LXRα and *S100A8* expression

As shown in Supplementary Figure S3, in the DEGs associated with ALI, *NR1H3* was significantly downregulated whereas *S100A8* was markedly upregulated, indicating an inverse correlation between LXRα and *S100A8*.

## Discussion

It is well known that LXRs, which are classified into LXRα and LXRβ subtypes, play important roles in various pulmonary diseases, including lung inflammation, asthma, ALI, and pulmonary fibrosis (Hernández-Hernández et al., 2024; Zhang et al., 2021; Yan et al., 2025; Shichino et al., 2019). In light of the significant roles of LXRα in cholesterol and lipid metabolism, inflammation, apoptosis, autophagy, and cellular bioenergetics (PPARγ) (Jalil et al., 2019; Ricote et al., 2004; Graham and Allen, 2015; Yang et al., 2020; Chen et al., 2024b; Dixon et al., 2021) as well as the bioinformatics-based finding that LXRα is significantly downregulated in the sepsis-related lung injury mouse model, we mainly focused on the regulatory effects of LXRα on the inflammatory responses in sepsis-induced ALI. Our experiments involved use of the synthetic agonist T0901317 (T0) to pharmacologically activate LXRα expression and demonstrate the effects of LXRα on ALI to explore the underlying mechanisms. In this study, we revealed that T0 protects against sepsis-related ALI via the following mechanisms: the total protein in the BALF was decreased; TNFα and IL6 mRNA expression levels were decreased in animals and cells; H&E staining revealed that the degree of lung injury was improved (Figure 3). These findings are consistent with the results of previous studies that endogenous LXR signaling prevents lung inflammation (Hernández-Hernández et al., 2024).

Autophagy represents a fundamental biological mechanism where autophagosomes engulf the cytoplasm or harmful invaders and subsequently fuse with lysosomes for degradation. This vital process is not only crucial for cellular maintenance and rejuvenation but also recognized as a key regulator of inflammatory responses and immune functions (Matsuzawa-Ishimoto et al., 2018; Deretic et al., 2015). Autophagy is ubiquitous in various biological systems and is important for facilitating cellular renewal, managing inflammatory reactions, and defending against pathogens (Mizushima and Komatsu, 2011). Previous studies have highlighted the connection between autophagy and inflammation in pulmonary diseases (Racanelli et al., 2018). It has been shown that autophagy in alveolar macrophages is crucial for suppressing spontaneous pulmonary inflammatory responses (Kanayama et al., 2015). Macrophage autophagy mainly reduces lung injury, and the mechanisms by which autophagy protects against lung injury are as follows: (1) inhibition of NLRP3 inflammasome activation (Peng et al., 2021); (2) reduction in endoplasmic reticulum stress levels (Fan et al., 2016); (3) suppression of MAPK1, MAPK8, and mTOR signaling (Liu et al., 2018; Li et al., 2022); (4) utilization of the PI3K/AKT signaling pathway (Qu et al., 2019). Research has shown that a deficiency related to the autophagy-associated proteins can exacerbate neutrophilic inflammation and lead to severe lung injury in mice (Suzuki et al., 2016; Pu et al., 2017). These findings indicate that autophagy plays a protective role in the host’s defense against acute pulmonary infections.

However, excessive autophagy may have negative effects and potentially contribute to ALI in the later stages of inflammation. An overabundance of autophagosomes could turn normal cellular protective responses into harmful ones (Lo et al., 2013; Tran et al., 2014; Chen et al., 2015). In smokers with chronic obstructive pulmonary disease (COPD), the role of autophagy is still debatable for pulmonary inflammation. Although some studies suggest that autophagy activation is detrimental to lung epithelial cells exposed to smoke (Lam et al., 2013; Mizumura et al., 2014; Wang et al., 2017), others report that reduced autophagic flux in the alveolar macrophages in smokers can impair bacterial delivery to the lysosomes, causing autophagy/lysosomal functional deficiency (Monick et al., 2010). Overall, the impacts of autophagy on pulmonary inflammatory diseases seem to depend on the stage of the disease and cell types involved in autophagy. In the present study, we performed bioinformatics analysis on a database and found that LXRα was distributed mainly in the macrophages, epithelial cells, and fibroblasts and was closely associated with autophagy (Figure 2); therefore, we selected macrophages to explore the mechanisms by which LXRα regulates sepsis-related lung injury by regulating macrophage autophagy. Our *in vitro* experiments revealed that T0 promoted LC3B expression and enhanced autophagy in sepsis-related ALI models. After autophagy was inhibited using 3-MA, the protective effects of T0 against inflammation decreased when challenged with LPS (Figures 4, 5). These findings suggest that LXRα protects against sepsis-related ALI by enhancing macrophage autophagy.

Previous studies have shown that autophagy is regulated by multiple genes, such as *ULK1/2*, *Atg5*, *Atg7*, and *mTOR* (Racanelli et al., 2018; Pareek and Kundu, 2024; Kinsella et al., 2023). To identify the key genes by which LXRα regulates autophagy in sepsis-induced lung injury, we performed RNA sequencing of RAW264.7 cells. We then intersected the upregulated and downregulated DEGs with an autophagy gene set to obtain four candidate genes, namely *ABCG1*, *FASN*, *S100A8*, and *SNORD118*. However, among these, only *S100A8* was increased in ALI and markedly decreased after T0 treatment, exhibiting a negative correlation with T0; the remaining three genes showed no clear relationships with T0. It has been reported that *S100A8*, which is an autophagy regulator (Yi et al., 2022), is involved in the suppression of LPS-induced lung inflammation (Fan et al., 2024; Yu et al., 2023), indicating that *S100A8* might be an important molecule regulated by LXRα. Our *in vitro* experiments further demonstrated that T0 suppressed the *S100A8* pathway. To further explore the clinical relationship between LXRα and *S100A8* expression in sepsis-induced ALI, we conducted a bioinformatics analysis and found an inverse correlation.

**FIGURE 6:**
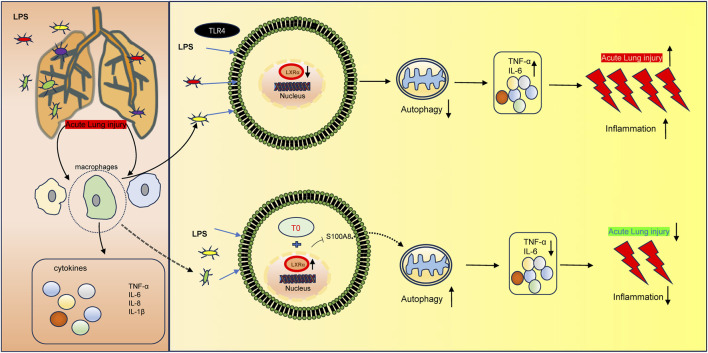
Molecular mechanism of LXR-mediated LPS-induced ALI development. LXRα expression is downregulated in LPS-induced ALI, thereby triggering an inflammatory response. Moreover, the upregulated LXRα could inhibit *S100A8* to increase autophagy, resulting in ALI.

In conclusion, the present study demonstrates that T0 enhances LXRα and further protects against sepsis-induced lung injury via *S100A8*, which is a component of the autophagy-related pathway (Figure 6). These results indicate that LXR/S100A8 may be a potential target for treating sepsis-related lung injuries. However, the present study has a major limitation. The specific molecular mechanisms by which LXRα regulates *S100A8* (e.g., whether LXR directly binds to the *S100A8* promoter through transcriptional regulation or indirectly regulates it via other pathways) is unclear. In the future, these could be investigated through ChIP-qPCR or dual-luciferase reporter gene experiments.

## Conclusion

The results of the present study show that LXRα is a critical negative regulator of sepsis-induced ALI. The pharmacological activation of LXRα with the synthetic agonist T0901317 markedly attenuated pulmonary inflammation, reduced alveolar protein leakage, and improved the histopathological scores in both murine and cellular models of ALI. Mechanistically, T0 exerted its protective effects by promoting macrophage autophagy, as evidenced by increased LC3-II accumulation and autophagosome formation, with simultaneous suppression of the autophagy-related gene *S100A8*. Inhibition of autophagy with 3-MA abolished the anti-inflammatory benefits of T0, while siRNA-mediated silencing of *S100A8* enhanced the anti-inflammatory benefits of T0, confirming that LXRα-mediated autophagy and *S100A8* downregulation are indispensable for lung protection. Furthermore, the results of integrated bioinformatics and human transcriptome analyses revealed a strong inverse correlation between LXRα and *S100A8* expression, underscoring the clinical relevance of this pathway. Collectively, our findings delineate a novel LXRα/S100A8 pathway that can be therapeutically targeted to mitigate sepsis-induced lung injury.

## Data Availability

The datasets presented in this study can be found in online repositories. The names of the repository/repositories and accession number(s) can be found in the article/Supplementary Material.

## Supplementary material

Supplementary Figure S1 Flow diagram of the present research.

Supplementary Figure S2 Cells were transfected with S100A8-siRNA (600 nM) and control-siRNA. After 6 h, the cells were co-incubated with lipopolysaccharide (LPS; 10 µg/mL) and harvested 6 h later. **(A)** Quantitative real-time polymerase chain reaction (qRT-PCR) analysis of the S100A8 mRNA levels in RAW264.7 cells (^#^ *p* < 0.05, control-siRNA+LPS vs. S100A8-siRNA+LPS). **(B)** TNFα mRNA levels in RAW264.7 cells (^#^ *p* < 0.05, control-siRNA+LPS vs. S100A8-siRNA+LPS). The results are presented in terms of mean ± standard error of the mean (SEM) values (n = 3).

Supplementary Figure S3 Clinical correlation analysis of LXRα and S100A8 expressions: **(A)** gene expression profiles; **(B)** volcano plot illustrating the differentially expressed genes (DEGs) between acute lung injury (ALI) and normal persons using the *limma* method; **(C)** bar chart illustrating NR1H3 and S100A8 gene expressions between ALI and normal persons; **(D)** correlation analysis between NR1H3 and S100A8 genes (R= −0.98, *p* < 0.001).

Supplementary Figure S4 NF-kBp65 expression in RAW264.7 cells detected by Western blotting: **(A)** NF-kB p65 protein levels were examined in RAW264.7 cells via Western blotting; **(B)** quantification of NF-kB p65 protein bands (^#^ *p* < 0.05, LPS vs. T0+LPS).
