# Supplementary figures and images for: Liver X receptor agonist T0901317 alleviates sepsis-induced acute lung injury by enhancing macrophage autophagy
Source: Front Pharmacol. 2025 Sep 9;16:1552034. doi: 10.3389/fphar.2025.1552034 (PMC12454352; doi:10.3389/fphar.2025.1552034)

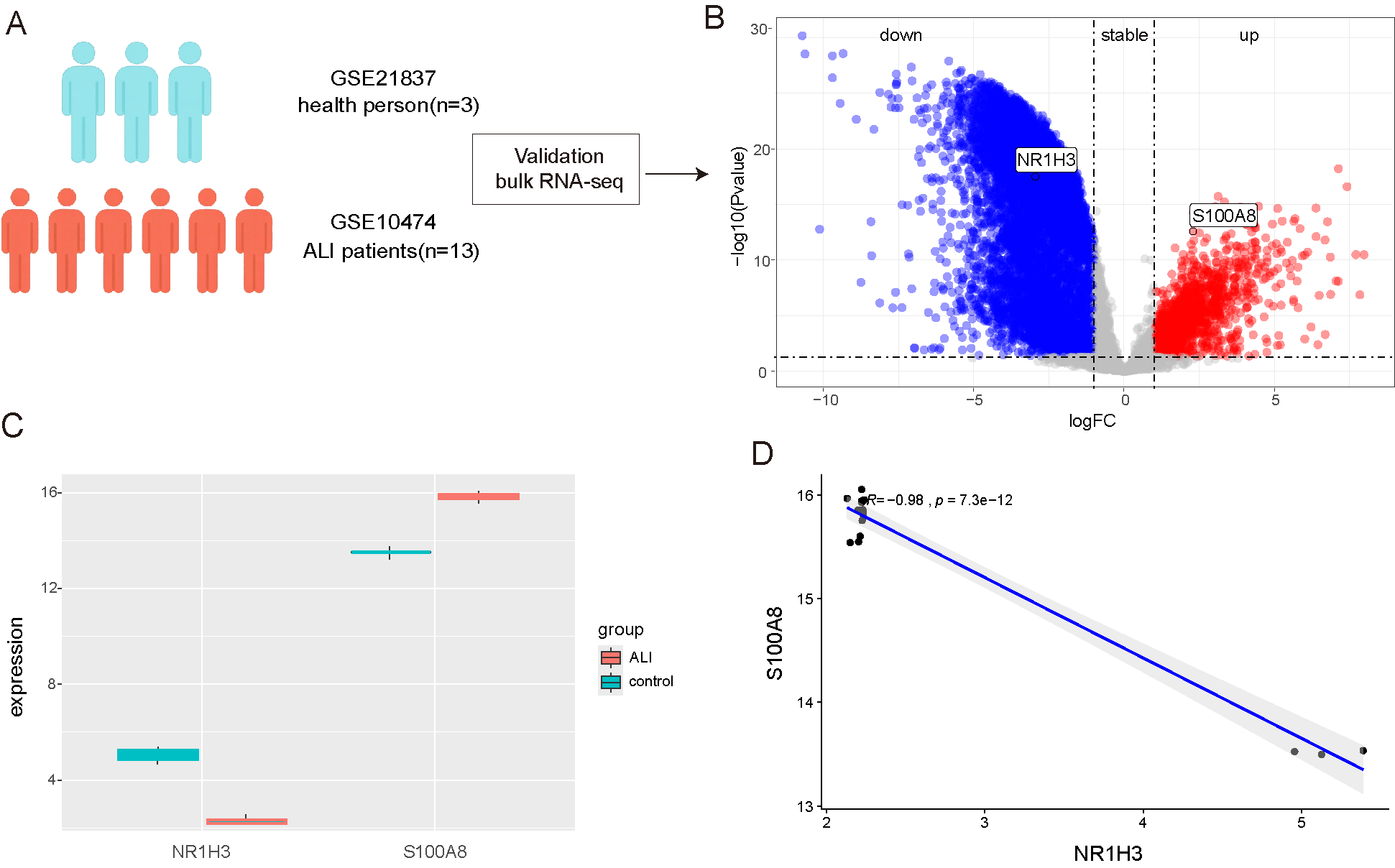

Supplement: Supplementary file 3 [file Image3.tif]

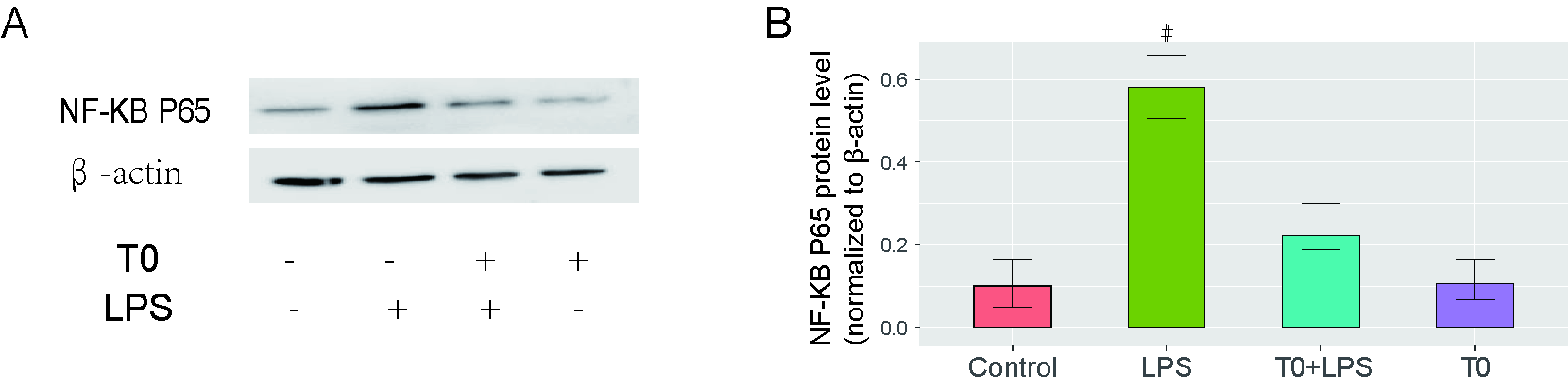

Supplement: Supplementary file 4 [file Image4.tif]

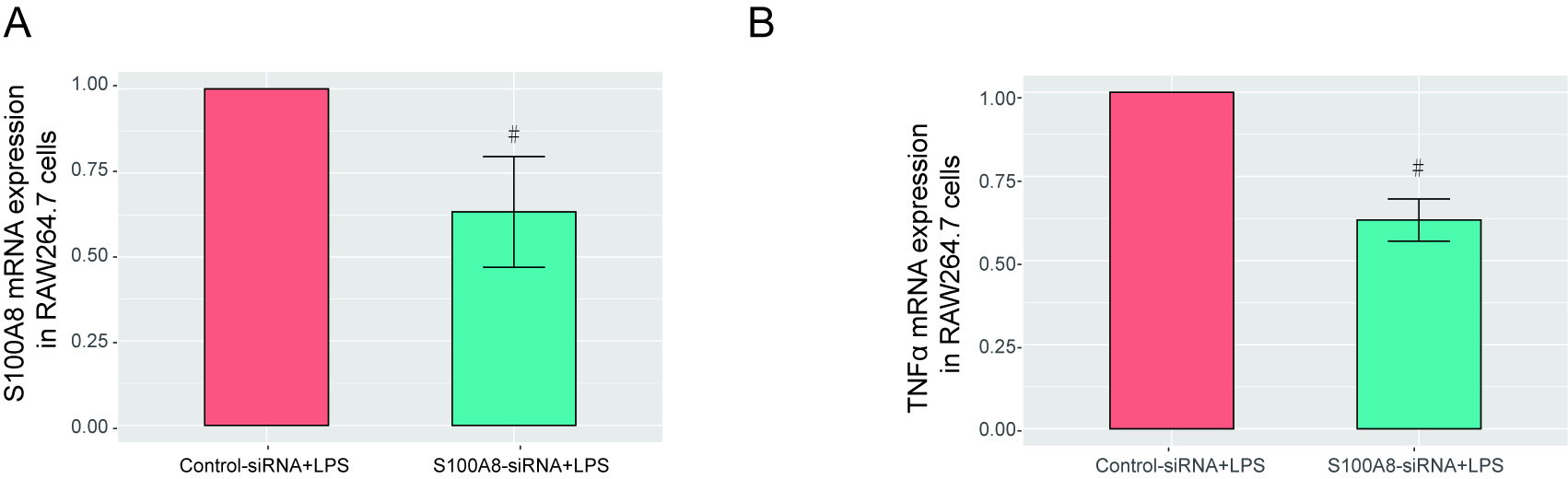

Supplement: Supplementary file 5 [file Image2.tif]

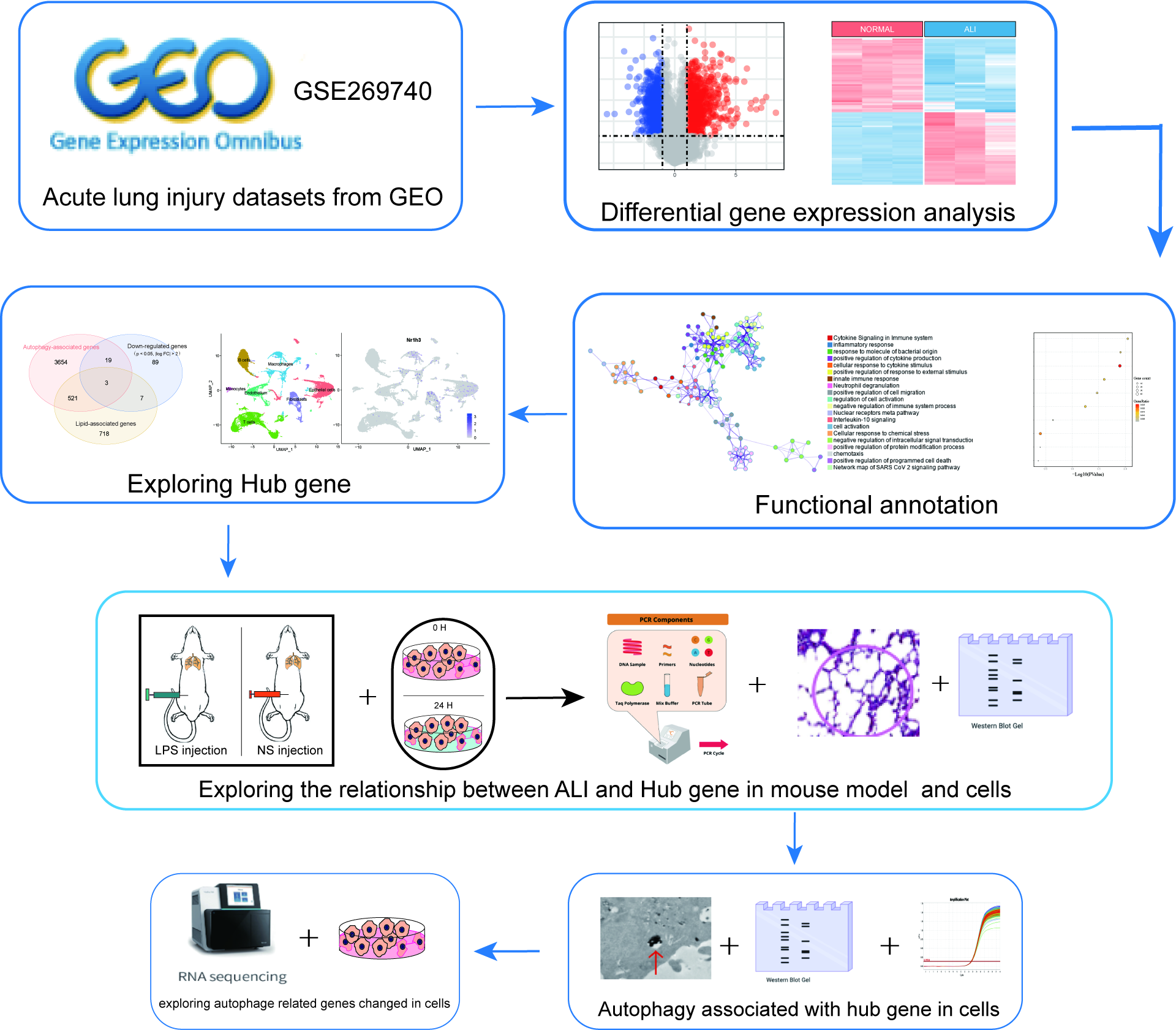

Supplement: Supplementary file 6 [file Image1.tif]
